# The Cellular Phenotype of Roberts Syndrome Fibroblasts as Revealed by Ectopic Expression of ESCO2

**DOI:** 10.1371/journal.pone.0006936

**Published:** 2009-09-07

**Authors:** Petra van der Lelij, Barbara C. Godthelp, Wouter van Zon, Djoke van Gosliga, Anneke B. Oostra, Jûrgen Steltenpool, Jan de Groot, Rik J. Scheper, Rob M. Wolthuis, Quinten Waisfisz, Firouz Darroudi, Hans Joenje, Johan P. de Winter

**Affiliations:** 1 Department of Clinical Genetics, VU University Medical Center, Amsterdam, The Netherlands; 2 Department of Toxicogenetics, Leiden University Medical Center, Leiden, The Netherlands; 3 Division of Molecular Biology, Netherlands Cancer Institute, Amsterdam, The Netherlands; 4 Department of Pathology, VU University Medical Center, Amsterdam, The Netherlands; Mount Sinai School of Medicine, United States of America

## Abstract

Cohesion between sister chromatids is essential for faithful chromosome segregation. In budding yeast, the acetyltransferase Eco1/Ctf7 establishes cohesion during DNA replication in S phase and in response to DNA double strand breaks in G2/M phase. In humans two Eco1 orthologs exist: ESCO1 and ESCO2. Both proteins are required for proper sister chromatid cohesion, but their exact function is unclear at present. Since *ESCO2* has been identified as the gene defective in the rare autosomal recessive cohesinopathy Roberts syndrome (RBS), cells from RBS patients can be used to elucidate the role of ESCO2. We investigated for the first time RBS cells in comparison to isogenic controls that stably express V5- or GFP-tagged ESCO2. We show that the sister chromatid cohesion defect in the transfected cell lines is rescued and suggest that ESCO2 is regulated by proteasomal degradation in a cell cycle-dependent manner. In comparison to the corrected cells RBS cells were hypersensitive to the DNA-damaging agents mitomycin C, camptothecin and etoposide, while no particular sensitivity to UV, ionizing radiation, hydroxyurea or aphidicolin was found. The cohesion defect of RBS cells and their hypersensitivity to DNA-damaging agents were not corrected by a patient-derived ESCO2 acetyltransferase mutant (W539G), indicating that the acetyltransferase activity of ESCO2 is essential for its function. In contrast to a previous study on cells from patients with Cornelia de Lange syndrome, another cohesinopathy, RBS cells failed to exhibit excessive chromosome aberrations after irradiation in G2 phase of the cell cycle. Our results point at an S phase-specific role for ESCO2 in the maintenance of genome stability.

## Introduction

Roberts syndrome (RBS) is a rare autosomal recessive disease characterized by growth retardation and congenital abnormalities. RBS patients typically have limb malformations involving symmetric reduction in the number of digits, and the length or presence of bones in the arms and legs, but the severity of these abnormalities is quite variable, even within families [1]. Survival is generally poor, as most cases of RBS end in spontaneous abortion, still-birth, or neonatal death. Cells from RBS patients show specific cytogenetic characteristics, mainly consisting of metaphase chromosomes displaying repulsion at heterochromatin regions or centromere splitting leading to a railroad-track appearance of chromosomes. RBS is caused by mutations in *ESCO2* [2]. ESCO2 is one of the two human orthologs of the *Saccharomyces cerevisiae* protein Eco1/Ctf7, a putative acetyltransferase required for the establishment of sister chromatid cohesion during S phase [3], [4]. In addition, Eco1 is important to maintain sister chromatid cohesion after the introduction of double strand breaks in G2/M phase of the cell cycle [5]–[7], suggesting that the establishment of cohesion is also essential for postreplicative repair of double strand breaks.

EBV-immortalized lymphoblastoid cell lines from Roberts syndrome patients have previously been claimed to be hypersensitive to the growth-inhibiting effect of mitomycin C (MMC) and gamma irradiation [8], [9]. However, these studies were not entirely conclusive since isogenic control cell lines were not available or not included. Gordillo *et al* showed that a lymphoblastoid cell line from a patient homozygous for the missense mutation W539G in the acetyltransferase domain of ESCO2 was as sensitive to MMC as lymphoblasts from RBS patients lacking ESCO2 mRNA and protein due to nonsense or frameshift mutations [8], indicating that the ESCO2 acetyltransferase domain is important for its function. This missense mutation reduced the acetyltransferase activity of ESCO2 *in vitro*, which suggests that it is actually pathogenic. Nevertheless, to explore the function of the human ESCO2 protein, there is a need for functionally corrected cell lines from Roberts syndrome patients.

In this study we investigate ESCO2-deficient immortalized skin fibroblasts from a Roberts syndrome patient in comparison to isogenic ESCO2 complemented cell lines to document the cellular phenotype of ESCO2-deficient cells. Cells lacking functional ESCO2 appeared to be characterized by a chromatid cohesion defect and by hypersensitivity to the DNA-damaging agents mitomycin C, camptothecin, and etoposide.

## Results

### Immortal fibroblasts from a Roberts syndrome patient are functionally complemented by epitope-tagged ESCO2

**Figure 1:**
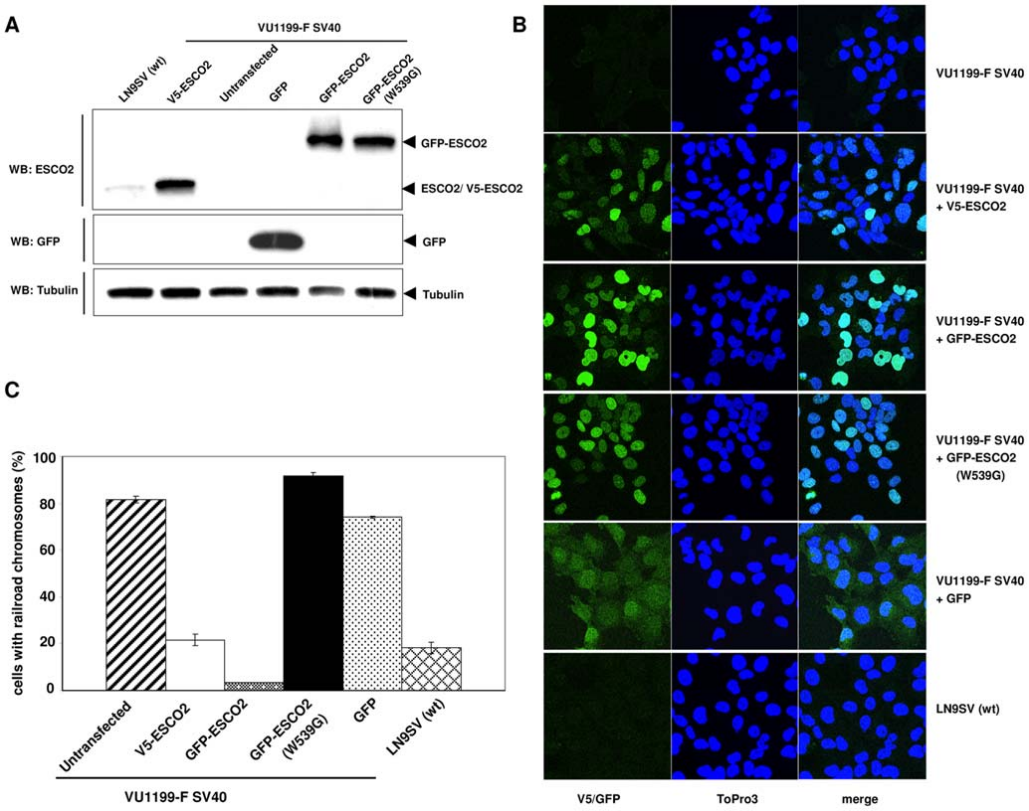
Functional complementation of Roberts syndrome fibroblasts by epitope tagged ESCO2. Stable VU1199-F SV40 cell lines expressing V5- or GFP-tagged ESCO2 were obtained by transfection and neomycin selection. (A) Whole cell extracts were analyzed for ESCO2 or GFP expression by Western blotting with an ESCO2- or GFP-specific antibody. Tubulin served as a loading control. The VU1199-F SV40 cell line stably transfected with a GFP construct served as a control for cells expressing GFP protein only. (B) Nuclear localization of V5-ESCO2, GFP-ESCO2 and GFP-ESCO2 (W539G) in the stably transfected VU1199-F SV40 fibroblasts. Cells were fixed with 4% methanol-free formaldehyde solution and the V5-ESCO2 expressing cell line was probed with an anti-V5 antibody. Nuclei were stained with ToPro3. (C) Railroad chromosomes in RBS immortal fibroblasts and complemented cell lines. Fifty metaphases per cell line were scored for the presence of railroad chromosomes, from coded slides; the percentage of metaphases containing one or more railroad chromosome was calculated.

To be able to study the role of ESCO2 in sister chromatid cohesion and DNA damage response, SV40 immortalized, ESCO2-deficient fibroblasts from a Roberts syndrome patient (VU1199-F SV40) were stably transfected with cDNA constructs encoding either V5- or GFP-tagged ESCO2 protein. As a negative control, a patient-derived mutation in the acetyltransferase domain of GFP-ESCO2 was generated (GFP-ESCO2 (W539G)). Upon neomycin selection, several clones were obtained, which expressed the V5-ESCO2 or GFP-ESCO2 proteins at levels much higher than endogenous ESCO2 in wild type fibroblasts (Figure 1A). As expected from their molecular weights GFP-ESCO2 ran more slowly in the SDS-PAGE gel than V5-ESCO2; Western blotting with a GFP-specific antibody was used to demonstrate that the GFP signal was exclusively derived from the GFP-ESCO2 fusion protein, since no uncoupled GFP molecules were detected (Figure 1A).

Both wild type and mutant ESCO2 proteins localized in the nucleus, where ESCO2 is supposed to perform its function, but the expression levels seemed to vary between cells (Figure 1B). Since overexpression and/or the V5 or GFP tags may interfere with the activity of ectopically expressed proteins, we tested functional complementation by cytogenetic analysis. Metaphase spreads revealed a strong reduction in the number of railroad chromosomes in cell lines stably transfected with wild type ESCO2 (Figure 1C), demonstrating that the V5- and GFP-tagged ESCO2 proteins are indeed functional, in spite of their relatively high expression levels. The occurrence of railroad chromosomes was not corrected by GFP alone nor by the mutant ESCO2 protein (Figure 1C). Given the relatively normal subcellular distribution of the mutant ESCO2 protein compared to the ectopically expressed wild type protein, our data indicate that the pathogenic character of missense mutation W539G is not due to mislocalization or instability of ESCO2, but most likely through its effect on the acetyltransferase activity of the protein.

These stable cell lines can now serve as isogenic controls for the characterization of the cellular phenotype of RBS fibroblasts and can be used to study the function of ESCO2 and its acetyltransferase domain in more detail.

### ESCO2 is regulated during the cell cycle by proteasomal degradation

**Figure 2:**
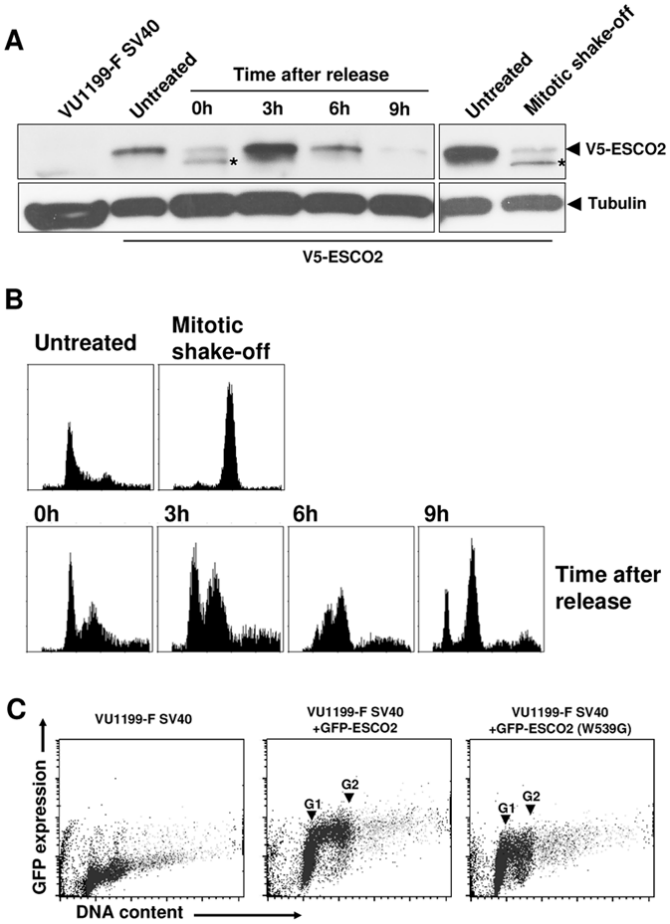
Ectopic ESCO2 expression during the cell cycle. VU1199-F SV40 cells stably expressing V5-ESCO2 were synchronized by a double aphidicolin block and mitotic shake-off and samples were analyzed for (A) ESCO2 expression on Western blot and (B) DNA content by flow cytometry. (C) Cell cycle distribution related to GFP-ESCO2 expression was measured by flow cytometry in VU1199-F SV40 cells stably expressing wild type or mutant GFP-ESCO2. Cells were fixed in 70% ethanol and DNA was stained with ToPro3. Asterisk indicates a protein detected by the ESCO2 antibody which is supposed to represent a degradation product of ESCO2.

A striking intercellular variation in ESCO2 protein expression was observed during long-term culture (Figure 1B), which suggested a cell cycle-dependent regulation of ESCO2. To test this possibility, we synchronized V5-ESCO2-expressing RBS cells with the replication inhibitor aphidicolin and followed ESCO2 expression after release from this cell cycle block (Figure 2A). At three hours post-treatment, when many cells were in S phase (Figure 2B), ESCO2 levels were high, while nine hours after release most cells were in G2/M phase and ESCO2 expression was barely detectable. Also in M phase cells obtained by mitotic shake-off V5-ESCO2 expression was very low. Flow cytometery of RBS cells expressing either wild type or mutant GFP-ESCO2, again showed a high ESCO2 expression in S phase cells, although the mutant appeared to be less stable (Figure 2C). Furthermore, time-lapse movies demonstrated a cell cycle regulated expression of GFP-ESCO2, with a very strong accumulation in the nucleoli in both untreated cells (Supplementary Video S1) and cells treated with the replication inhibitor hydroxyurea (Supplementary Video S2). Although so far we have not observed any other ectopically expressed protein to accumulate in the nucleolus, including GFP, we can not rule out that overexpression of tagged ESCO2 underlies its nucleolar localization, since our ESCO2 antibody is unable to detect endogenous ESCO2 protein by immunofluoresence. These data show that, despite of the CMV promoter-driven expression of ESCO2, protein levels vary during the cell cycle, possibly by posttranslational modification.

**Figure 3:**
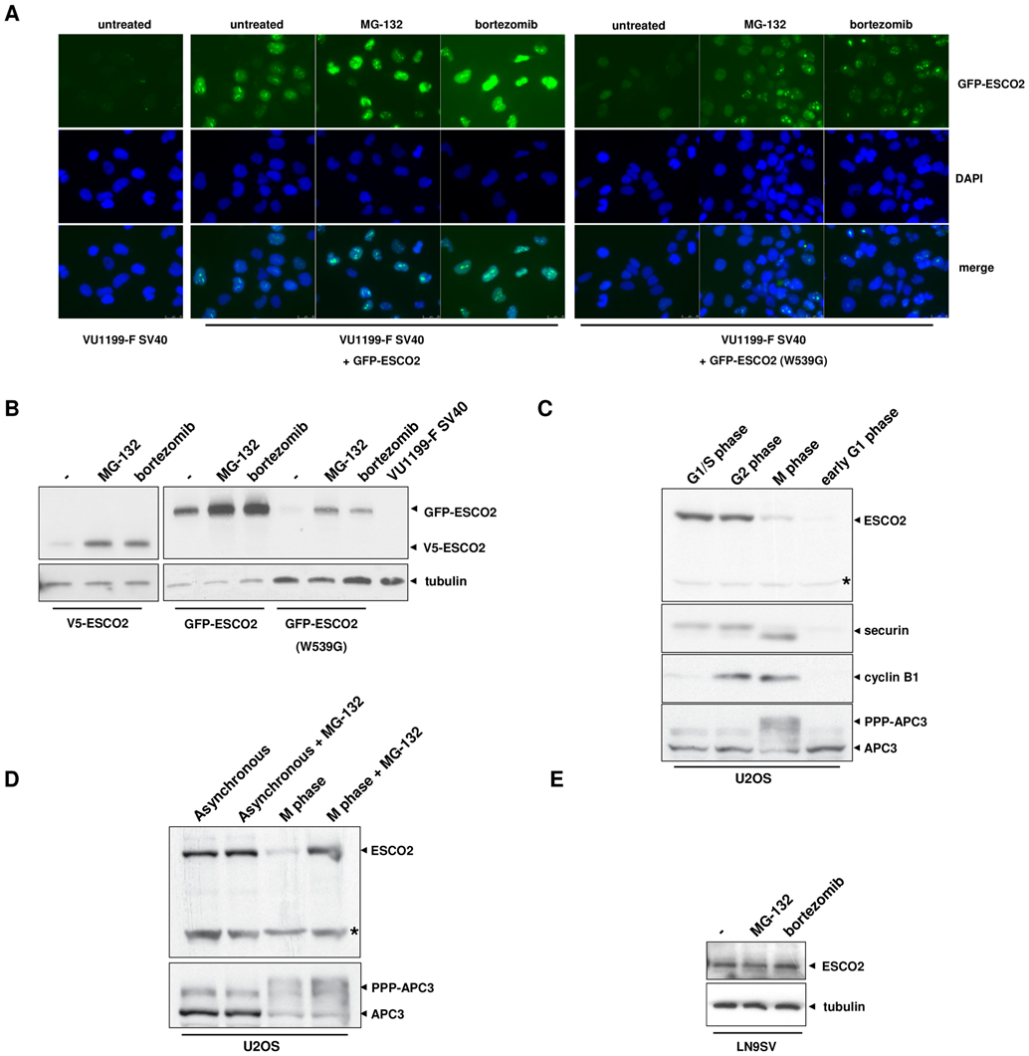
ESCO2 levels are regulated by proteasomal degradation. (A) Fluoresence microscopy showing stabilization of ESCO2 protein by proteasome inhibitors in VU1199-F SV40 cells stably expressing wild type or mutant ESCO2. Cells were treated for 6 h with 50 µM MG-132 or 100 nM bortezomib and fixed with 100% cold methanol. Nuclei were stained with DAPI. (B) Ectopic ESCO2 is stabilized by proteasome inhibitors as shown by Western blotting. Cell lines were exposed to proteasome inhibitors for 6 h with 50 µM MG-132 or 100 nM bortezomib and analyzed for ESCO2 expression by Western blotting. Tubulin was used as loading control. (C) Endogenous ESCO2 levels in synchronized U2OS cells. Cells were arrested in G1/S phase by a thymidine block and released for 9 h to obtain cells in G2 phase. Mitotic cells were harvested by treatment with nocodazole followed by mitotic shake-off. These cells were released for 90 min to obtain early G1 phase cells. Cell lysates were analyzed for ESCO2, securin, cyclin B1 and APC expression by Western blotting. Asterisk indicates an aspecific band considered as loading control. (D) Proteasome inhibitor MG-132 stabilizes ESCO2 levels in M phase cells. U2OS cells were cultured for 4 h in the presence of 5 µM MG-132 before M phase cells were isolated. APC3 phosphorylation is shown as a control for mitotic cells. Asterisk indicates an aspecific band as loading control. (E) ESCO2 levels in unsynchronized wild type fibroblasts treated with proteasome inhibitors. LN9SV was treated for 6 h with 50 µM MG-132 or 1 µM bortezomib.

The Western blot of synchronized V5-ESCO2 expressing cells (Figure 2A) revealed an ESCO2 protein with faster mobility (lanes 3 and 8, asterisks), which suggests proteolysis. One possible way to vary ESCO2 protein levels during the cell cycle is by regulated protein degradation through the proteasome. To explore this possibility, GFP-ESCO2 transfected RBS cells were treated with the proteasomal inhibitors MG-132 and bortezomib. Immunofluorescence clearly showed enhanced nuclear and nucleolar staining of wild type and mutant ESCO2 upon proteasome inhibitor treatment (Figure 3A). In addition, as indicated by Western blotting, the proteasome inhibitors increased the total level of wild type and mutant GFP-ESCO2 as well as that of V5-ESCO2 (Figure 3B). These data indicate that ectopic ESCO2 levels are regulated by proteasomal degradation, which is independent of its acetyltransferase activity.

Endogenous ESCO2 levels in U2OS cells also varied during the cell cycle with very low levels in M and early G1 phase cells compared to G1/S or G2 phase cells (Figure 3C). The production of ESCO2 preceded that of cyclin B1, which starts to rise during G2 phase and this is in agreement with a role for ESCO2 in S phase. ESCO2 is down-regulated in mitotic cells characterized by heavily phosphorylated APC3/cdc27, and this precedes a decrease in securin levels. Securin is a known target of the Anaphase-Promoting Complex/Cyclosome (APC/C), which is involved in the degradation of proteins during mitosis and these data suggest that ESCO2 and securin are regulated in different manners. Indeed, we did not observe stabilization of ESCO2 levels in mitotic extracts of cells treated with siRNA targeting APC/C subunits (data not shown). However, proteasome inhibitor MG-132 stabilized ESCO2 levels in mitotic cells (Figure 3D), indicating that also endogenous ESCO2 levels are regulated by proteasomal degradation. Proteasome inhibitors did not detectably affect ESCO2 levels in unsynchronized U2OS cells (Figure 3D) or wild type fibroblasts (Figure 3E).

In summary, our data indicate that both endogenous and ectopically expressed ESCO2 levels are regulated during the cell cycle by APC-independent proteasomal degradation.

### Hypersensitivity of Roberts syndrome cells to various DNA-damaging agents

**Figure 4:**
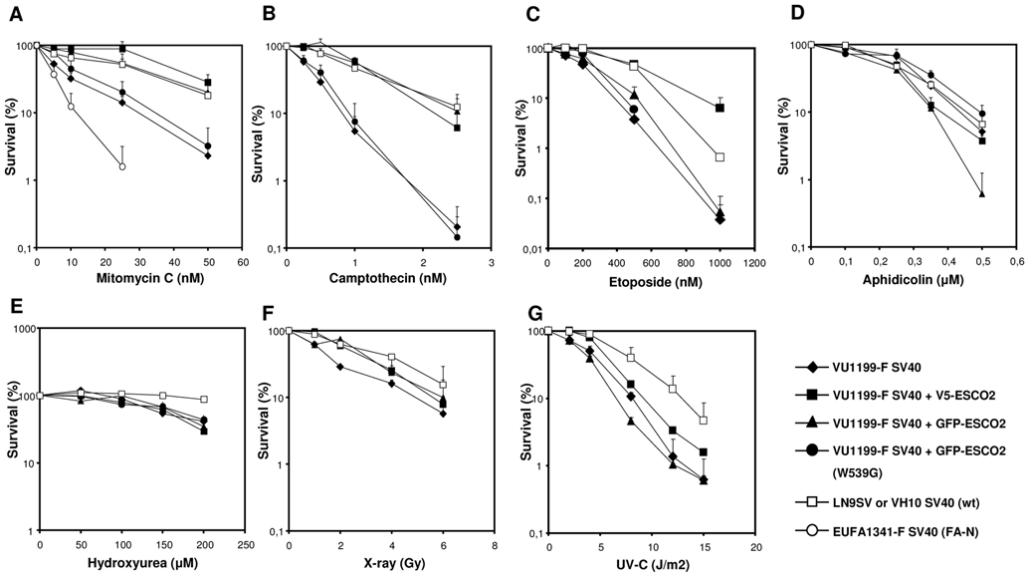
Survival of RBS and stably transfected fibroblasts after treatment with various DNA-damaging agents. Data are averages of at least 2 or 3 independent experiments; error bars represent standard error of the mean. VU1199-F SV40 cell line, V5-ESCO2-transfected VU1199-F SV40, GFP-ESCO2-transfected VU1199-F SV40, GFP-ESCO2 mutant (W539G)-transfected VU1199-F SV40 and wild type fibroblasts LN9SV (figures A to E) or VH10 SV40 (figures F and G) were grown for 10–12 days after treatment with X-rays or UV-C irradiation, or after continuous exposure to the indicated DNA-damaging agents. (A) Clonogenic survival after continuous MMC exposure. SV40-immortalized fibroblasts of a Fanconi anemia patient (EUFA1341, FA-N) are shown as a hypersensitive control (open circles). Clonogenic survival after continuous exposure to (B) camptothecin, (C) etoposide, (D) aphidicolin, (E) hydroxyurea. Clonogenic survival after (F) X-ray or (G) UV-C exposure.

Two previous studies have claimed that lymphoblastoid cells from Roberts syndrome patients are unusually sensitive to growth inhibition by the DNA cross-linking agent mitomycin C (MMC), which interferes with DNA replication [8], [9]. To exclude the influence of differences in growth rate and possible heterogeneity of cell lines, we used colony survival assays to determine the MMC sensitivity of cell line VU1199-F SV40 and its isogenic control lines stably expressing V5- or GFP-tagged ESCO2. RBS fibroblasts exposed to increasing doses MMC showed reduced survival when compared to the isogenic V5-ESCO2- or GFP-ESCO2-expressing RBS fibroblasts, as well as compared to non-isogenic wild type fibroblasts (Figure 4A), confirming the MMC hypersensitivity of ESCO2-deficient cell lines. However, relative to Fanconi anemia fibroblasts, the sensitivity of RBS cells was less pronounced. In contrast to wild type ESCO2, the ESCO2 acetyltransferase mutant was not able to correct the MMC sensitivity of VU1199-F SV40, indicating that the ESCO2 acetyltransferase domain is essential for cellular tolerance to MMC and that ESCO2 may play a role in DNA cross-link repair.

MMC is very effective in blocking DNA replication forks by covalently connecting the two complementary DNA strands, but many other agents are able to interfere with DNA replication. To further document the impact of ESCO2 deficiency on the cellular DNA damage response we tested several additional agents with different mechanisms of action, including camptothecin, etoposide, aphidicolin, hydroxyurea, X-rays and UV-C.

The topoisomerase I inhibitor camptothecin creates single-stranded DNA breaks by fixing the topoisomerase I-DNA cleavage intermediate on the DNA [10]. RBS fibroblasts showed a hypersensitivity to camptothecin when compared to their complemented counterparts or wild type fibroblasts (Figure 4B), whereas expression of the ESCO2 acetyltransferase mutant failed to rescue this hypersensitive phenotype.

DNA replication can also be disrupted by the topoismerase II inhibitor etoposide, which stabilizes a topoisomerase II-DNA complex and prevents religation of the DNA after the enzyme has generated a double strand break [11]. Also for this agent, colony survival assays revealed a hypersensitive phenotype in RBS fibroblasts, which was completely rescued by ectopic expression of V5-ESCO2 when compared to VU1199-F SV40 cells or fibroblasts expressing the ESCO2 acetyltransferase mutant (Figure 4C). Interestingly, in this particular instance the GFP-ESCO2 protein appeared as inactive as the acetyltransferase mutant, suggesting that the GFP-tag may interfere with the ability of ESCO2 to assist in the handling of etoposide-induced DNA damage. Although these data suggest a role for ESCO2 in the response to replication fork stalling, we did not find an increased sensitivity to the more general replication inhibitors aphidicolin or hydroxyurea, which inhibit replicative polymerase alpha or deplete the dNTP pool by inhibiting ribonucleotide reductase, respectively (Figure 4D and 4E). This indicates that ESCO2 is specifically involved in a response to DNA-damaging agents that physically block DNA replication forks.

To test whether cells from Roberts syndrome patients have a more general DNA repair defect we created double strand breaks by exposing the cell lines to several doses of X-rays. Colony survival assays showed no marked difference in X-ray sensitivity between RBS fibroblasts and the corrected cell lines. Similar results were found after UV treatment (Figure 4F and 4G). The lack of ionizing irradiation sensitivity may be explained by the fact that the exposure time is short and that only cells in a specific phase of the cell cycle are X-ray sensitive. Recently, it has been shown that, despite a lack of X-ray sensitivity in clonogenic survival, cells from Cornelia de Lange syndrome patients, another cohesinopathy, show a 2–4-fold increase in X-ray induced chromosomal aberrations than control cells when irradiated in the G2 phase of the cell cycle [12]. However, both immortal fibroblasts (Table 1) and primary fibroblasts (Table 2) from RBS patients do not show this increased sensitivity for G2-induced chromosomal aberrations. Together with the hypersensitivity to DNA-damaging agents that block DNA replication and the highly regulated protein expression levels, these data suggest a role for ESCO2 in genomic maintenance during the S phase of the cell cycle.

**Table 1:** X ray-induced chromosomal aberrations in SV40-immortalized human fibroblasts irradiated in the G2 phase of the cell cycle.

Cell line	X-ray dose (Gy)	Breaks/cell	Interchanges/cell	Total breaks/cell*	Induced breaks/cell**
VH10 SV40 (wt)	0	0.35	0	0.35	0
	0.25	0.60	0.02	0.64	0.29
	0.50	1.18	0.09	1.35	1.00
VU1199-F SV40 (RBS)	0	0.50	0.04	0.58	0
	0.25	0.90	0.04	0.98	0.40
	0.50	1.53	0.14	1.81***	1.23

* Frequency of total breaks estimated by counting one chromatid interchange as two breaks.

** Induced break frequency is estimated by subtracting values obtained in control from those obtained after irradiating cells.

*** One multi-aberrant cell was found among 100 cells analyzed.

**Table 2:** X-ray induced chromosomal aberrations in primary human fibroblasts irradiated in G2 phase of the cell cycle.

Cell line	X-ray dose (Gy)	Breaks/cell	Interchanges/cell	Total breaks/cell*	Induced breaks/cell**
VH10 (wt)	0	0.01	0	0.01	0
	0.1	0.10	0	0.10	0.09
	0.25	0.20	0	0.20	0.19
VU1174-F (RBS)	0	0.02	0	0.02	0
	0.1	0.10	0	0.10	0.08
	0.25	0.24	0	0.24	0.22

* Frequency of total breaks estimated by counting one chromatid interchange as two breaks.

** Induced break frequency is estimated by subtracting values obtained in control from those obtained after irradiating cells.

### Normal Rad51 focus formation and sister chromatid exchange in RBS cells

**Figure 5:**
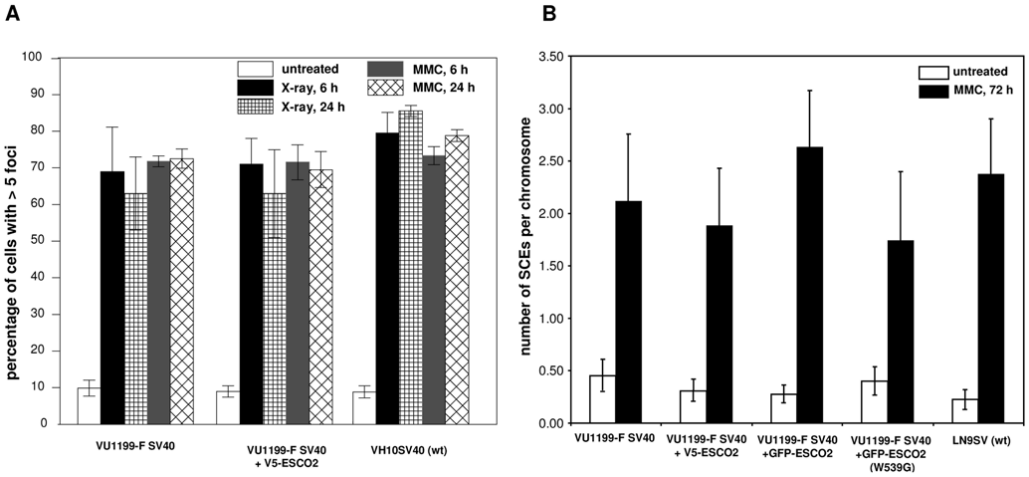
Formation of Rad51 foci and sister chromatid exchanges in RBS cells. (A) Rad51 foci in normal and RBS cells, as determined 6 and 24 h after treatment with X-ray (12Gy) or MMC treatment (7 µM for 1 h). The percentages of cells containing more than five nuclear foci were determined. Data are the means of at least three experiments; error bars represent the standard error of the mean. (B) SCE induction in RBS cells after MMC treatment. Wild type (LN9SV), RBS (VU1199-F SV40) and RBS cells expressing V5-ESCO2, GFP-ESCO2 or GFP-ESCO2 (W539G) were either mock-treated or treated by continuous exposure to 50 nM MMC. Numbers of SCEs were counted and normalized against the number of chromosomes scored.

The repair of DNA damage in S and G2 phase often involves homologous recombination and it is conceivable that cohesion between sister chromatids is essential for this process. A key protein in homologous recombination repair is Rad51, which localizes in nuclear foci after DNA damage is induced. To investigate the role of ESCO2 in the relocalization of Rad51, the formation of Rad51 foci was studied in RBS cells. As shown in Figure 5A, RBS cells were capable of forming Rad51 foci in response to X-ray irradiation or MMC treatment with kinetics comparable to wild type fibroblasts, which indicates that ESCO2 is not directly involved in the homologous recombination process.

A fraction of homologous recombination events results in cross-over recombination between sister chromatids, which can be visualized by differential staining of sister chromatids during DNA replication, so-called sister chromatid exchanges (SCEs). When using this method to analyze the number of sister chromatid exchanges in RBS cells no marked abnormalities in spontaneous or MMC-induced SCEs were found (Figure 5B). This confirmed the notion that ESCO2 is not essential for homologous recombination.

### Increased Chk1 phosphorylation in RBS cells

**Figure 6:**
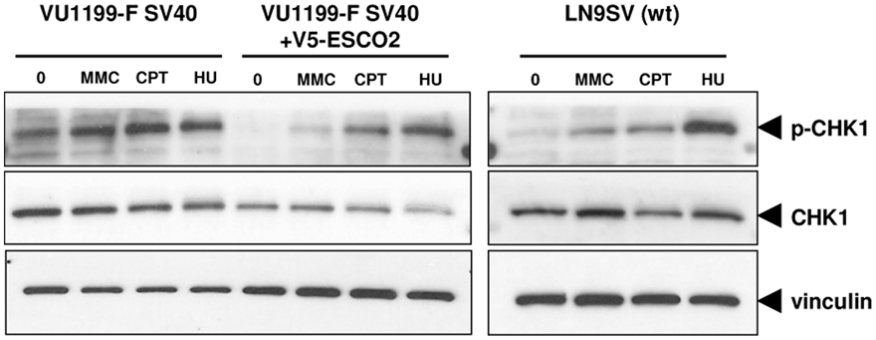
Chk-1 phosphorylation status in RBS cells. VU1199-F SV40 fibroblasts, VU1199-F corrected with V5-ESCO2 and wild type fibroblasts LN9SV were left untreated or exposed to MMC, camptothecin (CPT) or hydroxyurea (HU) for 24 h and whole cell extracts were obtained to investigate the phosphorylation of Chk-1. Vinculin and Chk-1 served as loading controls.

Stalled replication forks activate the DNA damage kinase ATR. A downstream target of ATR is Chk1, which is phosphorylated upon replication fork stalling. Western blotting of whole cell extracts revealed that RBS fibroblasts have increased levels of phosphorylated Chk1 as compared to functionally corrected RBS fibroblasts and wild type fiboblasts (Figure 6). In addition, the induction of Chk1 phosphorylation by MMC, camptothecin and HU appears to be less pronounced in RBS cells. These results suggest the occurrence of excessive levels of replication-coupled DNA damage in RBS fibroblasts.

## Discussion

Roberts syndrome is a rare autosomal recessive multisystem developmental disorder that is caused by mutations in *ESCO2* [2]. Clinical features are highly variable and have been described in great detail [1], but little is known about the cellular characteristics in RBS. Cell lines from RBS patients can be used to explore the role of ESCO2 in more detail. Here we demonstrate that ESCO2-deficient fibroblasts have defects in sister chromatid cohesion and show sensitivity to several DNA-damaging agents. These defects can be corrected by ectopic expression of wild type ESCO2, but not by a patient-derived ESCO2 acetyltransferease mutant (W539G), indicating a direct role for the acetyltransferase activity of ESCO2 in sister chromatid cohesion and the DNA damage response.

Establishment of sister chromatid cohesion during S phase of the cell cycle is required for faithful chromosome segregation in M phase. Studies in yeast have revealed that cohesion is mediated by the cohesin proteins Smc1, Smc3, Scc1 and Scc3 [13], which are loaded onto the chromatin by a complex of Scc2 and Scc4 before DNA replication starts [14]. The acetyltransferase Eco1/Ctf7, the yeast ortholog of ESCO2, is essential for the establishment of sister chromatid cohesion during S phase in a process that is closely linked to DNA replication [15], [16]. In addition, it was shown that, as a response to double strand breaks in G2/M phase, genome-wide cohesion is established by a replication-independent reactivation of Eco1 [6], [7]. This process depends on Mec1, the yeast ortholog of DNA damage kinase ATR, but also requires Scc2 [6], [7], which loads the cohesin complex in G2/M phase irrespective of DNA damage [15]. Recently, Smc3 was reported as the first *in vivo* substrate of Eco1 [17]–[19]. However, siRNA knockdown experiments in HeLa cells showed that not ESCO2, but ESCO1, another Eco1 ortholog, is the acetyltransferase required for SMC3 acetylation in human cells [19]. Mcd1/Scc1 has been proposed as an Eco1 substrate for damage-induced cohesion establishment in G2/M phase after Mdc1/Scc1 phosphorylation by Chk1, a downstream effector of Mec1 [20], but it is unclear whether this is mediated by ESCO1 or ESCO2.

Both ESCO1 and ESCO2 are essential for sister chromatid cohesion, and they are not functionally redundant [21], but only mutations in *ESCO2* have so far been found in RBS patients [2], [8], [22]. The homology between the ESCO1 and ESCO2 protein is remarkably restricted to the zinc finger and acetyltransferase domain. The similarity between ESCO1 and ESCO2 reaches 77% (59% homology) within this C-terminal domain, while no significant homology can be detected outside of this region [21]. Although both proteins have acetyltransferase activity, the diversity of their N-terminal domains suggests that they may perform distinct functions in the establishment of sister chromatid cohesion. This idea is supported by the differences in protein expression patterns seen throughout the cell cycle [21]. ESCO1 is present during the entire cell cycle, while ESCO2 expression is high during S phase and disappears in G2/M phase. This may imply that ESCO1 is involved in the establishment of sister chromatid cohesion in S, G2 and M phase, whereas ESCO2 may have a specific function in the establishment of sister chromatid cohesion during S phase. In our stable cell lines, ectopic ESCO2 protein levels also varied during the cell cycle, with high expression during S phase. Since in these cells ESCO2 mRNA expression is not under the control of its normal promoter, but driven by the CMV promoter, it is very likely that ESCO2 protein levels are regulated by posttranslational modification. The protective effect of proteasome inhibitors on ESCO2 levels strongly suggests that proteasomal degradation is indeed involved in this process.

We have used our stable cell lines to confirm that ESCO2 deficiency leads to cohesion defects and in addition demonstrated that this sensitizes cells for MMC, camptothecin and etoposide. These DNA-damaging agents interfere with DNA replication by covalently linking bases on opposite DNA strands (MMC) or by creating complex single strand (camptothecin) or double strand (etoposide) breaks. This indicates that ESCO2 functions in the repair process at the stalled replication fork. The lack of sensitivity to the replication inhibitors hydroxyurea and aphidicolin suggests that it is not the inhibition of DNA replication, but the replication-blocking DNA damage *per se* which causes a problem that RBS cells are less well able to handle.

ESCO2 seems to have a minor role in the repair of UV-C and X-ray induced DNA damage; however, this result could be misleading due to the fact that only cells in a specific phase of the cell cycle may be sensitive to these agents and that relatively few sensitive cells are hit if exposure is only of short duration such as with UV- and X-irradiation. To further investigate this we looked at X-ray induced chromosomal aberrations in cells irradiated in the G2 phase of the cell cycle. In Cornelia de Lange syndrome cells, which have a defect in cohesin loader SCC2, G2 cells show an increase in irradiation-induced chromosomal aberrations [12] as expected from the involvement of Scc2 in damage-induced cohesin loading in the G2 phase of yeast cells [6], [7]. In contrast, ESCO2-deficient cells do not show significantly more chromosomal damage in cells irradiated in the G2 phase of the cell cycle. This again underlines the S phase-restricted role for ESCO2 and suggests that ESCO1 may be the Eco1 ortholog that plays a role in damage-induced cohesion establishment during G2 phase. The lack of X-ray sensitivity in our fibroblasts is in conflict with previous data in lymphoblasts [9]. The discrepancy could be due to differences between cell types, but phenotypic heterogeneity in the lymphoblastoid cell lines could also explain this dissimilarity, since isogenic controls were not used in the lymphoblast study.

During S and G2 phase homologous recombination is the preferred pathway for double strand break repair and the formation of sister chromatid exchanges (SCEs) or the appearance of Rad51 foci after DNA damage can both serve as indicators for intact homologous recombination repair. SCEs reflect sites of homologous recombination that are associated with crossing-over between sister chromatids [23], while Rad51, a central player in homologous recombination, shows a punctuated nuclear localization at sites where homologous recombination takes place [24], [25]. RBS cells were able to form SCEs at similar levels as corrected control cells. Also, Rad51 foci after DNA damage were formed to similar extents in RBS and control cells. These results suggest that the establishment of sister chromatid cohesion by ESCO2 is not essential for homologous recombination in general. Our data do not exclude a role for ESCO2 in regional or site-specific regulation of the homologous recombination process. We detected a strong accumulation of ESCO2 in the nucleolus upon replication fork stalling, which may indicate a role for ESCO2 at repetitive sequences like rDNA. This may not be reflected in an overall decrease in SCEs or Rad51 foci, but may become critical when replication is disturbed by DNA-damaging agents. Of note here is that RBS fibroblasts were less sensitive to MMC than fibroblasts from a PALB2 deficient Fanconi anemia patient, which has a more general defect in crosslink repair and shows a disturbance in the formation of Rad51 foci [26]. Regional or site-specificity of ESCO2's role during S phase may also explain the apparent hyper-phosphorylation of Chk1 in untreated RBS cells.

Based upon our results and previously published studies a model can be put forward, in which ESCO2 is essential for the cohesion establishment at the replication fork through acetylation of substrates that remain to be identified. Acetylation of SMC3 by ESCO1 is also important in this process, but how this modification relates to ESCO2-mediated acetylation remains to be established. In the G2 phase cohesion loading by SCC2 and SMC3 acetylation through ESCO1 may be involved in a replication-independent process of cohesion establishment. The identification of ESCO1 as an ATM/ATR substrate [27] and the requirement of the yeast ATR ortholog Mec1 for cohesion establishment in G2/M phase [6], [7] strengthen this hypothesis.

Together our results indicate that ESCO2 is an important acetyltransferase involved the establishment of sister chromatid cohesion and the maintenance of genomic stability during S phase. RBS fibroblasts overexpressing wild type ESCO2 and ESCO2 mutants as described in this paper are important tools to further explore the role of ESCO2 in these processes.

## Materials and Methods

### Ethics statement

The research on patient material was carried out after approval by the Institutional Review board of the VU Medical Center that adhered to local ethical standards, and was initiated only after the relevant informed consents had been obtained [2].

### Cell culture

Primary skin fibroblasts from a two-months old male Roberts syndrome patient homozygous for the mutation 877_878 delAG in exon 4 (reported in [2]) were immortalized by transfection with a plasmid encoding the SV40 large-T antigen. Several weeks after transfection colonies of transformed cells appeared, which were mixed and further propagated. The transformed cells have been in continuous culture for over 60 passages and were therefore considered immortal. This immortalized RBS cell line (VU1199-F SV40), primary RBS fibroblasts (VU1174-F), an immortalized fibroblast cell line from a Fanconi anemia (complementation group N) patient with a PALB2 defect (EUFA1341-F SV40) and wild type cell lines (LN9SV and VH10 SV40) were cultured in Ham's F10 medium (Gibco, Paisley, UK) supplemented with 10% fetal bovine serum (FBS, Hyclone, Logan, USA). Stable cell lines were generated by transfection of *Pvu*I linearized expression vector pIRESneo containing cDNAs encoding GFP, V5- or GFP-tagged wild type ESCO2, or a GFP-tagged ESCO2 acetyltransferase mutant (W539G). These stable cell lines were cultured in complete medium containing G418 at 150 µg/ml (Calbiochem, Nottingham, UK). Human osteosarcoma (U2OS) cells were cultured in DMEM supplemented with 8% fetal calf serum, penicillin (100 U/ml) and streptomycin (100 µg/ml).

### Plasmid constructs

Full-length ESCO2 cDNA was PCR-amplified from wild type lymphoblasts with a forward cDNA primer containing an *Xho*I restriction site and a reverse primer containing a *Bam*HI restriction site. The PCR fragment was subcloned in pBluescript SK^-^ (Stratagene, La Jolla, USA) and the sequence was verified. A Kozak sequence and V5-tag were added to the 5′ end of the construct by PCR and subcloned into the pBluescript construct using the *Xho*I site and an internal *Hind*III site. The PCR product was again sequenced to check the integrity of the construct. The cDNA was then subcloned in the *Xho*I and *Bam*HI restriction sites of mammalian expression vector pCEP4 (Clontech, Mountain View, USA) and finally transferred to mammalian expression vector pIRESneo3 (Clontech) using *Not*I and *Bam*HI.

To generate a cDNA construct encoding GFP-ESCO2, full-length ESCO2 cDNA was PCR amplified from the pIRESneo3+V5-ESCO2 vector with a forward cDNA primer containing an *Xho*I restriction site and a reverse primer containing a *Bam*HI restriction site. The PCR fragment was subcloned into pEGFP-C1 (Clontech) to create a GFP-ESCO2 fusion construct. The GFP-ESCO2 cDNA was subcloned to pIRESneo3 using *Nhe*I and *Bam*HI, after which the sequence was verified.

The patient-derived missense mutation W539G was generated by PCR using a forward cDNA primer covering the internal *Pst*I restriction site in ESCO2 in which the mutation was introduced and a reverse primer containing a *Bam*HI restriction site. The *Pst*I-*Bam*HI fragment from wild type ESCO2 in pBluescript SK^-^ was replaced by this PCR fragment and the construct was sequenced. An internal *Eco*RI restiction site and the *Bam*HI restriction site were used to replace the wild type sequence in pIRESneo3 GFP-ESCO2 by the mutant sequence.

A pIRESneo plasmid containing GFP was generated by subcloning cDNA encoding GFP from pEGFP-C1 into pIRESneo by the use of *Bam*HI and *Nhe*I restriction sites.

A construct for the purification of a GST-ESCO2 fusion protein was generated in the prokaryotic expression vector pGEX-KG. A cDNA fragment encoding ESCO2 amino acids 216 to 359 was obtained by PCR on ESCO2 cDNA using primers with *Bam*HI and *Eco*RI restriction sites. This fragment was subcloned in the *Bam*HI and *Eco*RI sites of pGEX-KG.

### Generation of ESCO2 antiserum

A His-tagged ESCO2 fusion protein representing amino acids 217 to 359 was obtained from MorphoSys (Martinsried, Germany) and used to immunize guinea pigs. GST-ESCO2_217–359_ was expressed in *E. coli* and purified as described [28]. The purified GST-ESCO2 fusion protein was immobilized on an AminoLink Plus column (Pierce, Rockford, USA) according to the manufacturer's instructions. This column was used to affinity-purify the ESCO2 antiserum, as described [29].

### Synchronization of cells

For synchronization experiments, VU1199-F+V5-ESCO2 cells were treated with a double aphidicolin block. Cells were seeded, treated with 10 µM aphidicolin (Sigma, St. Louis, USA) for 16 h, followed by an 8 h release period after which they were treated with 10 µM aphidicolin for another 16 h. For release, aphidicolin was washed away and cells were cultured in normal medium. Synchronized cells were isolated by cell scraping at different time points. Mitotic cells were obtained by mitotic shake-off after treatment with 100 ng/ml nocodazole (Sigma) for 18 h. Cells were divided for Western blot analysis and flow cytometry. Cell cycle profiles were obtained by permeabilization in buffer containing 100 mM Tris-HCL (pH 7.5), 150 mM NaCl, 0.5 mM MgCl_2_, 1 mM CaCl_2_, 0.2% BSA and 0.1% NP-40, followed by staining of DNA with 0.1 µg/ml Hoechst 33258 (Sigma). DNA content was measured with a Partec PAS flow cytometer (Görlitz, Germany) and analyzed with Flowmax software (Partec). VU1199-F + GFP-ESCO2 cells were analyzed on a BD FACScalibur (BD Biosciences, San Jose, USA) for GFP expression and DNA content after fixation in 70% ethanol and DNA staining with 1 µM ToPro3 (Invitrogen).

U2OS cells were used to analyze the levels of endogenous ESCO2 at different phases of the cell cycle. Cells were synchronized at the G1 to S phase transition with thymidine (2.5 mM for 24 h) and in G2 phase by releasing the cells from the G1/S block for 9 hours. Addition of the spindle poison nocodazole (250 ng/ml for 12 h), after release from the thymidine block, arrested cells in mitosis. Subsequently, mitotic cells were collected by mitotic shake off, of which half were allowed to leave mitosis by washing away the nocodazole and replating the cells in fresh medium for 90 minutes, allowing cell division and entry into early G1 phase. The synchronized cells were lysed in ELB buffer (50 mM HEPES (pH 7.5), 50 mM NaCl, 0.3% NP-40, 5 mM EDTA, 6% glycerol, 10 mM β-glycerophosphate, 5 mM NaF, 1 mM Na2VO3, and protease inhibitor cocktail (Roche)).

### Proteasome inhibition

ESCO2 expression levels upon proteasome inhibition were measured 6 h after incubation with either 50 µM MG-132 (Sigma) or 100 nM bortezomib (VUmc pharmacy). Cells were fixed with 100% methanol and nuclei were stained for 15 min with 1 µg/ml DAPI for fluorescence microscopy, or scraped in PBS, resuspended in sample buffer (10% glycerol, 60 mM Tris-HCL pH 6.8, 2% SDS, 0.002% bromophenol blue) and sonicated for analysis on Western blot.

### Western blot analysis

Whole-cell extracts were prepared in lysis buffer (50 mM Tris (pH 7.4), 150 mM NaCl, and 1% Triton X-100 supplemented with protease and phosphatase inhibitors). To examine the Chk1 phosphorylation status, cells were treated for 24 h with 100 nM MMC (Kyowa Hakko Kogyo Co., Tokyo, Japan), 5 nM camptothecin (Sigma), or 1 mM hydroxyurea (Sigma). Cells were scraped on ice in lysis buffer and sonicated. Proteins were separated on 8%–16% Tris-glycine gradient gels (Invitrogen, Eugene, USA) and transferred to Immobilon-P membrane (Millipore, Billerica, MA). Membranes were blocked with 5% dry milk in TBST (10 mM Tris HCL pH 7.5, 150 mM NaCl, 0.05% Tween-20) and probed with the purified guinea pig antiserum against ESCO2_217–359_ (1∶1000), rabbit-anti GFP (Clontech, 1∶100), rabbit-anti phospho-Chk1 (S317) (1∶500, Bethyl laboratories, Montgomery, USA), mouse-anti Chk1 (1∶500, Santa Cruz Biotechnology, Santa Cruz, USA), mouse-anti vinculin (1∶1000, Santa Cruz Biotechnology), mouse-anti tubulin (1∶20,000, Abcam, Cambridge, UK), rabbit anti-Securin (1∶200, Zymed, San Francisco, USA ), mouse anti-cyclin B1 (GNS1, 1∶200, Santa Cruz Biotechnology) or mouse anti-APC3/Cdc27 (1∶1000, BD Transduction Laboratories, San Jose, USA)). After washing with TBST, the membranes were incubated with peroxidase-conjugated goat immunoglobulins (DAKO, Glostrup, Denmark). Proteins were visualized with the ECL Western blotting detection system (Amersham Biosciences, Piscataway, USA).

### Immunofluorescence microscopy

To examine the cellular localization of the ESCO2 protein, cells were grown on sterile chamber slides (Nunc, Roskilde, Denmark). Cells were fixed with 4% methanol-free formaldehyde solution (Thermo Scientific, Waltham, USA). V5-ESCO2 expressing cells were blocked with 10% FBS in phosphate-buffered saline (PBS), incubated for 1 h at RT with an anti-V5 monoclonal antibody (1∶200, Invitrogen) followed by washing and incubation with AF488-labeled secondary anti-mouse antibody (1∶2000, Invitrogen) in the dark. All cell lines were incubated with ToPro3 for nuclear staining (1 µM, Invitrogen). After washing with PBS, the chamber slides were mounted on cover slides with Vectashield mounting medium (Vector Laboratories, Burlingame, USA). Cells were examined using a Confocal Laser Scanning Microscope (Carl Zeiss). To examine Rad51 foci formation, fibroblasts were grown on sterile glass slides, resulting in sub-confluent cells at the time of fixation. Cells were either mock-treated or treated with MMC (7 µM for 1 h) or X-ray irradiation (5 or 12 Gy) and stained as described before [12].

### Time-lapse fluorescence microscopy

Stable VU1199-F SV40 GFP-ESCO2 cells were plated on 35 mm glass-bottom culture dishes (Willco-dish, Amsterdam, The Netherlands) and transferred to a heated culture chamber (37°C, 5% CO_2_) on a Zeiss Axiovert 200M microscope, equipped with a 0.55 numeric aperture condenser and a 40X Plan-Neo DIC objective (N.A. 1.3). A Photometrics Coolsnap HQ CCD camera (Scientific, Tucson, USA) with a GFP/DsRed dual band pass filter set (Chroma Technology Corp. Rockingham, USA) was used to visualize specific fluorescence. Images were processed using MetaMorph software (Universal Imaging, Downington, USA). To arrest cells in S phase, cells were treated with 1 mM hydroxyurea.

### Railroad chromosomes and SCEs in metaphase spreads

Cells were grown to ∼80% confluence. After 40 minutes demecolcin treatment (200 ng/ml, Sigma), cells were harvested, incubated with 0.075 M KCl for 20 minutes at room temperature, and fixed with 75% methanol, 25% acetic acid. Cells were dropped on a slide, air-dried, and stained for 5 minutes in a 4% Giemsa solution (Merck). For each culture, 50 metaphases were scored for the presence railroad chromosomes, from coded slides. The percentage of metaphases with ≥1 railroad chromosome was determined. For SCEs, cells were either mock-treated or treated with 50 nM MMC and 5-bromodeoxyuridine (BrdU, 1 µg/ml, Sigma) was added to the medium for 72 h. For fast-growing LN9SV cells, BrdU was added to the medium forty-eight hours prior to fixation. During this culture period, incorporation of BrdU into replicating cells allows for the identification of second-division metaphases showing differentially stained chromatids. Cells were fixed and dropped on glass slides, as described above. UV-irradiation followed by Giemsa staining was used to visualize SCEs. The frequency of SCEs was calculated by dividing the number of sister chromatid exchanges observed by the number of chromosomes scored.

### Assessment of G2-radiosensitivity

For G2 chromosome aberration analysis, exponentially growing immortalized fibroblasts (VH10 SV40 or VU1199-F SV40) and primary fibroblasts were irradiated with doses of 0, 0.1, 0.25 and 0.5 Gy. Following irradiation colcemid was added to the cultures, and cells were fixed after three hours, as described [12]. Preparations were made and stained with 2% aqueous Giemsa-solution and air-dried. For each dose, 50–150 metaphases were examined for chromosomal damage. The induced break frequency was calculated by subtracting values obtained in untreated cells from values obtained in radiation-treated cells.

### Clonogenic survival and growth inhibition assays

Exponentially growing cells were trypsinized and 500–2000 cells were plated in 9-cm dishes, in duplicate, and irradiated or exposed continuously to different doses of MMC, camptothecin, etoposide (Sigma), aphidicolin, or hydroxyurea in complete medium. After 10–12 days, the colonies were fixed with 75% methanol, 25% acetic acid, dried, and stained with 10% Giemsa solution, after which colonies were counted. In all experiments, wild type immortalized fibroblasts (LN9SV and VH10 SV40) were treated in an identical manner to serve as controls.

## Supporting Information

Video S1 GFP-ESCO2 expression increases during S phase in living cells. GFP-ESCO2 expression in GFP-ESCO2 corrected VU1199-F cells were followed through the cell cycle (time in hours:minutes)
(5.58 MB AVI)

Video S2 GFP-ESCO2 expression increases during S phase in living cells. GFP-ESCO2 expression in GFP-ESCO2 corrected VU1199-F cells were followed through the cell cycle treated with 1 mM hydroxyurea (Time in hours:minutes).
(9.66 MB AVI)
